# Orthodontic Treatment of a Transmigrating Impacted Lower Canine Using a Digitally Designed and 3D-Printed Lingual Appliance Combined with Corticotomy and Laser Therapy—A Case Report

**DOI:** 10.3390/jcm14041368

**Published:** 2025-02-19

**Authors:** Anna Ewa Kuc, Jacek Kotuła, Maria Kulgawczyk, Krzysztof Kotuła, Zuzanna Grzech-Leśniak, Aneta Zalewska, Justyna Kulikowska-Kulesza, Beata Kawala, Joanna Lis, Michał Sarul

**Affiliations:** 1 Department of Dentofacial Orthopedics and Orthodontics, Wroclaw Medical University, Krakowska 26, 50-425 Wroclaw, Poland; j_kotula@poczta.onet.pl (J.K.); beata.kawala@umw.edu.pl (B.K.); joanna.lis@umw.edu.pl (J.L.); 2 Dental Star Specialist Centre of Orthodontics, Krakowska 4/2, 15-875 Bialystok, Poland; kulgawczyk.m@gmail.com; 3 Medical Faculty, Pomeranian Medical University, Rybacka 1, 70-204 Szczecin, Poland; krzyskotula@gmail.com; 4 Faculty of Medicine and Dentistry, Wroclaw Medical University, Krakowska 26, 50-425 Wroclaw, Poland; zuzanna.grzech-lesniak@student.umw.edu.pl; 5 Aneta Zalewska Dentistry, Norwida 12, 15-196 Białystok, Poland; anetazal05@wp.pl; 6 Faculty of Law, University of Bialystok, Mickiewicza 1, 15-213 Bialystok, Poland; j.kulikowska@uwb.edu.pl; 7 Department of Integrated Dentistry, Wroclaw Medical University, Krakowska 26, 50-425 Wroclaw, Poland; michal.sarul@umw.edu.pl

**Keywords:** transmigration, canine impaction, corticotomy, photomodulation, 3D-printed appliance

## Abstract

**Background:** Canines play a vital functional and aesthetic role in human dentition, yet impacted canines, particularly in the mandible, are rare and can lead to functional disorders, such as the absence of canine guidance, while negatively affecting a patient’s self-esteem. Transmigration of mandibular canines adds complexity to treatment. One method to reduce the treatment time, especially for impacted teeth, is corticotomy-assisted orthodontic therapy (CAOT). **Methods:** A 13-year-old patient presented with a horizontally impacted lower right canine, positioned below the roots of the lower incisors, showing transmigration. A digitally designed and 3D-printed lingual appliance was attached to the lower molars with hooks on the lingual side, enabling the application of multi-directional orthodontic forces. CAOT was performed using an Er:YAG laser (LightWalker, Fotona, Ljubljana, Slovenia) at 200 mJ, 12 Hz, 2.4 W, with a pulse duration of 100 µs, and an MSP H14 conical tip (0.6 mm spot diameter). Photobiomodulation (PBM) with a 635 nm diode laser (Lasotronix, Smart ProM, Piaseczno, Poland) was applied at 10 J per point (20 J/cm^2^) for 100 s per point, with a total energy of 20 J per session to reduce the risk of root resorption, manage pain, and accelerate healing as the tooth was moved into the alveolar ridge. **Results:** The treatment duration was two and a half years. The lingual appliance with hooks allowed precise traction of the canine, aided by exposure from the lingual side and the attachment of a hook. Gentle forces applied via orthodontic thread gradually moved the canine beneath the oral mucosa. Mid-treatment cone beam computed tomography (CBCT) scans confirmed the absence of root resorption of the lower incisors. A corticotomy, enhanced by laser therapy, was performed before moving the canine into the alveolar ridge. The canine was successfully rotated 180° and positioned without any signs of resorption in the canine or adjacent teeth. **Conclusions:** The use of a digitally designed and 3D-printed lingual appliance with hooks allowed the precise control of the traction of impacted teeth. When combined with corticotomy and laser therapy, it minimised root resorption risks, reduced pain, accelerated healing, and improved the overall success of the orthodontic treatment.

## 1. Introduction

Dental anomalies, such as changes in their number, order of eruption, impaction in the bone, and changes in the structure or dimensions of teeth, often present significant challenges across various fields of dentistry [1].

Currently, one of the more common pathologies encountered by orthodontists is impacted teeth. A tooth is classified as impacted when it remains partially or completely covered by bone and mucous membrane for more than two years beyond the expected physiological eruption period [2]. The most commonly impacted teeth are mandibular third molars, maxillary canines, and mandibular premolars [3].

Impacted mandibular canines are particularly rare, with their incidence estimated to range from 0.92% to 1.35% [4]. Their occurrence is approximately 20 times less frequent than that of impacted maxillary canines [5].

A notable feature of impacted mandibular canines is their ability to transmigrate. The prevalence of mandibular canine transmigration is reported to range from 0.1% to 0.31% [6]. Transmigration is defined as the intraosseous pre-eruptive migration of a tooth across the midline [7,8,9].

The exact aetiology of transmigration remains unclear. However, it is hypothesised to be associated with certain pathological conditions, such as follicular cysts, odontomas, supernumerary teeth, or premature extraction of primary canines. In 2002, Mupparapu proposed a classification system for intraosseous transmigration and ectopic eruption of mandibular canines based on their migration patterns and positions relative to the mandibular midline, categorising them into five types as follows [10]:

Type I: Canines positioned mesially across the midline, either labially or lingually in relation to the anterior teeth. Type II: Horizontally impacted canines near the lower border of the mandible, below the apices of the incisors. Type III: Eruption of the canine on the opposite side. Type IV: Horizontally impacted canines near the lower border of the mandible, below the apex of the lateral teeth on the opposite side. Type V: Canines positioned vertically in the midline, with the long axis of the tooth crossing the midline.

Based on radiographs, the angle of inclination of the long axis of the unerupted canine can be determined, at the same time determining its possibility of transmigration. If this angle is in the range of 30° to 50°, there is a high probability that the tooth will cross the midline. However, if the angle of inclination exceeds 50°, transmigration becomes inevitable [5].

The process of canine eruption begins in the mixed dentition. The standard time of eruption of the mandibular canines falls between 9 and 10 years of age [7]. In the case of impacted permanent teeth, there are usually no clinical symptoms, except for the lack of eruption of the canine beyond the chronological age of eruption [3]. Impacted mandibular canines may sometimes be located on the lingual side of the alveolar process, which may then manifest as a palpable, hard swelling under the mucosa [5].

Canines play a vital functional and aesthetic role in human dentition. Impacted canines can contribute to functional disorders, such as the absence of canine guidance, and can adversely affect a patient’s self-esteem [1,11].

The aetiology of impacted teeth is diverse and multifactorial. Failure of tooth eruption into the oral cavity may be attributed to local, systemic, or genetic factors. Local factors include a lack of space in the dental arch, mesial migration of teeth due to premature loss of primary teeth, persistent primary teeth, trauma, and inflammatory or pathological changes. Systemic causes include anaemia, rickets, vitamin D deficiency, and endocrine disorders [2]. However, it is widely believed that the most common contributing factor is a discrepancy between the length of the dental arch and the size of the tooth [12].

Although most impacted teeth are asymptomatic, they can result in various complications, such as incorrect positioning of the impacted tooth, transmigration, migration of adjacent teeth, resorption of adjacent teeth, marginal bone resorption around the impacted tooth and adjacent teeth, cyst formation, and tumours [1,2,12].

Dental age is an important and frequently used parameter for estimating the timing of tooth eruption. Each specific group of teeth is expected to erupt at a particular time. However, this process can be influenced by local or systemic factors, which may lead to premature or delayed eruption. Consequently, dental age is not a particularly reliable method for predicting eruption timing. A more accurate approach involves the analysis of tooth development through radiographic imaging, which allows the precise assessment of tooth development. If radiographs reveal unerupted permanent canines that have reached their expected root length, the dental age corresponds to the chronological age. In such cases, if the roots of primary teeth have not sufficiently resorbed, it can be inferred that these primary teeth are impeding the proper eruption of permanent teeth [5].

Panoramic radiographs are a routine diagnostic tool in dentistry and serve as a basic imaging technique for identifying pathological changes and assessing impacted teeth. However, for a precise evaluation of the position of impacted canines, particularly in relation to adjacent teeth, and for a detailed assessment of any associated pathology, cone beam computed tomography (CBCT) is recommended [2,9,11,13].

Orthodontic treatment of impacted and transmigrating mandibular canines can be challenging. Awareness of the prevalence of these dental anomalies is essential for effective treatment planning and the management of potential complications [1]. The goal of treating impacted canines is to achieve correct alignment within the dental arch without adverse effects on the impacted tooth, adjacent teeth, or periodontal tissues. The diagnosis and management of impacted canines are often multidisciplinary, requiring a combination of orthodontic and surgical interventions [11]. The most common treatment modalities for impacted mandibular canines include surgical extraction with orthodontic closure of the resulting space, surgical exposure followed by orthodontic repositioning, auto-transplantation, or preservation of the primary canine, provided its root length and prognosis are favourable [3,14]. Wherever possible, interventional treatment should be initiated promptly after detecting the anomaly. Extraction of the primary canine may facilitate reorientation of an aberrantly positioned permanent canine [5].

Surgical exposure and orthodontic repositioning of an impacted tooth is a prolonged process. One method used to reduce the duration of orthodontic treatment, particularly in cases of impacted teeth, is Corticotomy-assisted orthodontic treatment (CAOT). CAOT is a modification of traditional corticotomy that integrates periodontal and orthodontic interventions. This technique involves the surgical decortication of the alveolar process, which entails deliberate cutting or disruption of the cortical bone, followed by bone grafting and orthodontic alignment. The outcomes of this procedure include minimising the risk of root resorption, reducing pain, and achieving long-term improvements in periodontal health. Additionally, it shortens the duration of orthodontic treatment and enhances its stability [15,16].

When planning treatment for impacted teeth, it is crucial to consider multiple factors, including the severity of the malposition, treatment duration, orthodontic biomechanics, periodontal stability, relationships with adjacent teeth, and the surgical techniques employed [14].

The introduction of new orthodontic procedures, particularly those using digital technologies and laser therapies, raises complex legal and regulatory issues related to medical devices and the practice of dentistry. In the European Union, 3D-printed dental appliances fall under Medical Device Regulation (MDR) 2017/745 [17], which places a number of stringent requirements on design validation, manufacturing processes, and clinical evaluation. This is the regulation that, among others, addresses customised-device considerations such as orthodontic appliances and requires the manufacturer to maintain design specifications, manufacturing processes, and post-market surveillance detailed records. Laser therapy application in dentistry is under both medical device regulations and professional practice guidelines on the qualification of practitioners, consent of patients, and safety protocols. Most national dental regulatory bodies have supplementary requirements to be met regarding the adoption of new technologies in orthodontic practice, which comprise obligatory training programmes and certification processes.

This article presents a proposed protocol that can be used in similar cases both to precisely apply force and to minimise the risk of resorption of adjacent teeth and the impacted tooth during insertion from the oral cavity through a lingual cortical plate into the alveolar ridge using a local corticotomy. It may be an alternative to extracting such a tooth.

Objective: Connecting the specific clinical methods used (e.g., 3D-printing, corticotomy, and laser therapy) with the broader orthodontic challenges of managing impacted and transmigrating canines.

## 2. Materials and Methods

**Figure 1:**
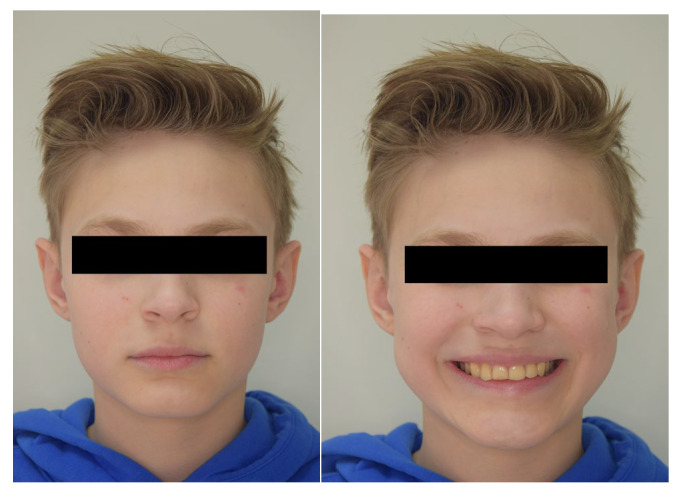

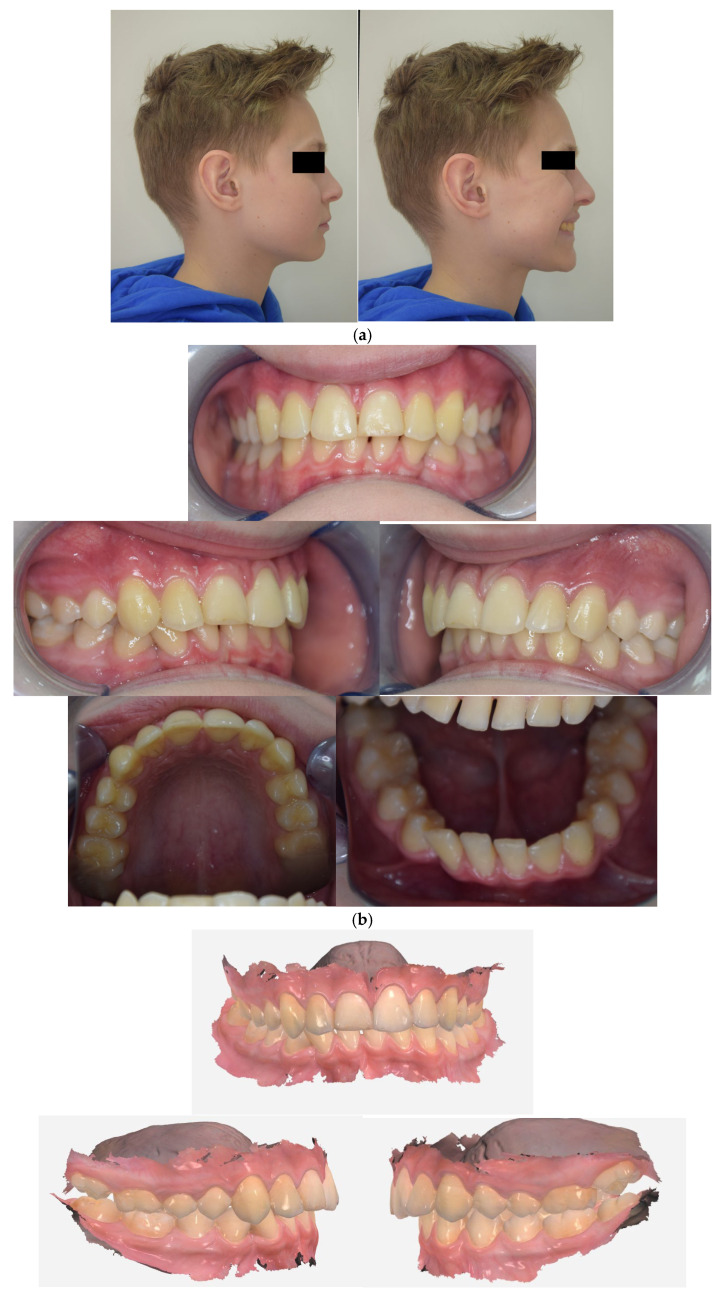

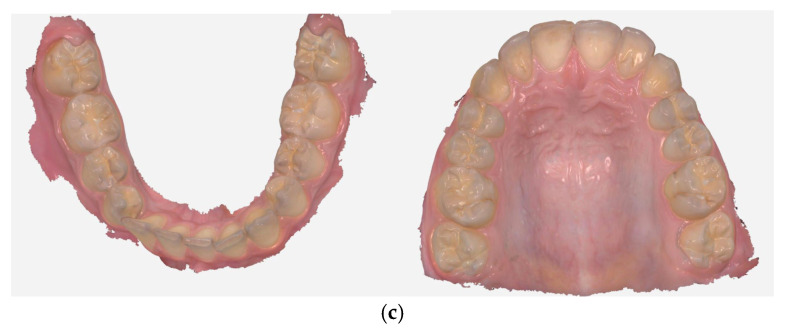
Photos before treatment: (**a**) extraoral, (**b**) intraoral and (**c**) models.

**Figure 2:**
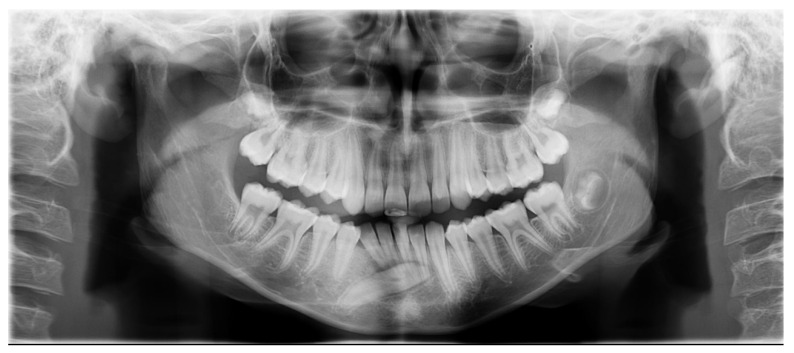

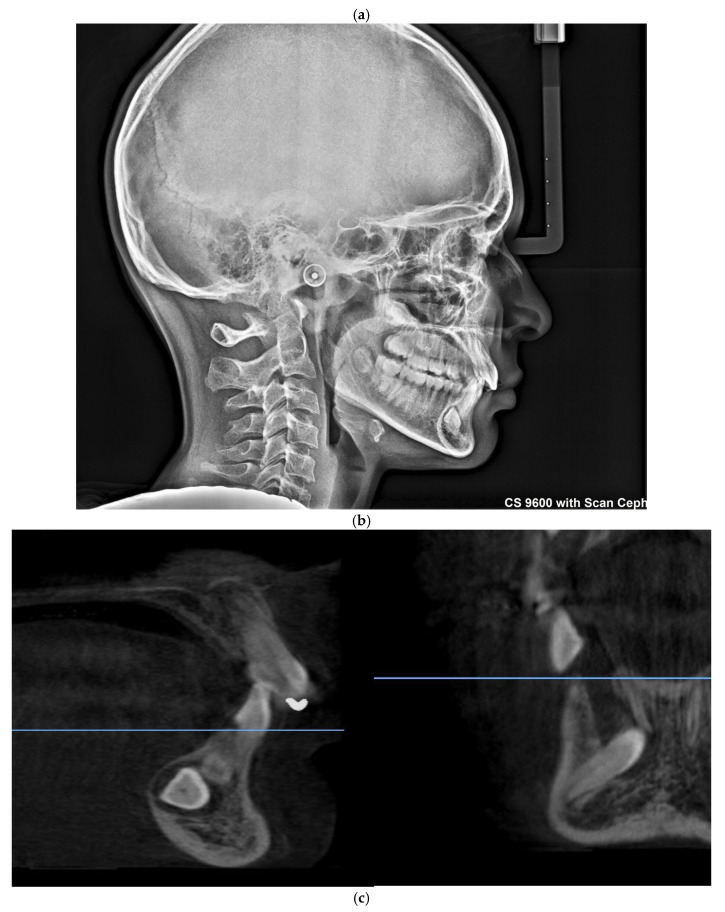
Panoramic radiograph (**a**), cephalometric (**b**), and CBCT (**c**) of a patient with a horizontally impacted right lower canine, located below the tips of the lower incisors, demonstrating transmigration.

A 13-year-old patient presented with a horizontally impacted lower right canine located beneath the apices of the lower incisors, exhibiting transmigration. The patient had Angle Class I occlusion with no significant dental abnormalities (Figure 1). Following a detailed analysis of the CBCT scan of the mandible (Figure 2), a decision was made to recreate space within the arch and reposition the impacted canine. Given the close proximity of the canine to the roots of the lateral incisors, precise orthodontic traction guidance was required.

**Figure 3:**
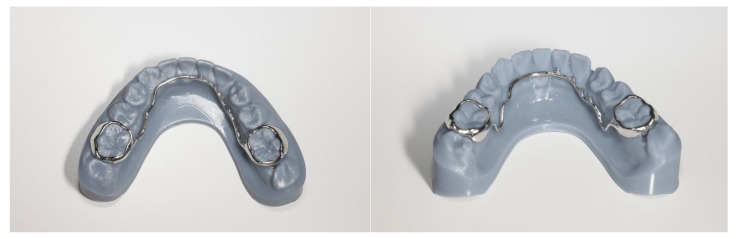
Digitally designed and 3D printed lingual arch with rings for lower first molars and hooks on the lingual side.

To achieve the desired direction of canine traction, a digitally designed and 3D-printed lingual arch was developed, featuring hooks on the lingual side to facilitate the application of multidirectional orthodontic forces (Figure 3). This appliance was used in conjunction with a conventional fixed orthodontic system.

The lingual arch with hooks for pulling down an impacted canine was created using CAD/CAM technology. This technology allows for precise design of the appliance and its adaptation to the patient’s physiological conditions. It increases the predictability of the treatment process by enabling more accurate planning of force application and vectors. Thanks to the individualised design and the integration of CBCT imaging, it is possible to plan the placement of the anchoring elements of the appliance on the teeth—custom rings and components to which elastic elements can be attached. The software integrates the hard tissues visible on the patient’s intraoral scans (teeth) with their counterparts in the tomography. As a result, the construction of the appliance is predictable, precise, and will not negatively impact soft tissues even during activation of its elements.

The use of CBCT imaging is particularly crucial for impacted teeth. When designing such work analogically, it is impossible to determine the distance between the attachment on the impacted tooth and the anchor points for elastic forces on the appliance structure.

The appliance was designed using 3Shape Appliance Designer software, version Trios 4, which is among the best available solutions. The many years of experience of 3Shape and the technical expertise of the laboratory ensure the proper design and execution of the appliance. The work was printed at a certified printing centre using Scheftner’s Starobond CoS Powder 55 alloy (S&S Scheftner GmbH, Mainz, Germany) (composition: Co 59%, Cr 25%, W 9.5%, Mo 3.5%, Si 1%, C, Fe, Mn, N < 1%).

After diagnostic procedures, the digitally designed appliance was attached to the lower first molars.

**Figure 4:**
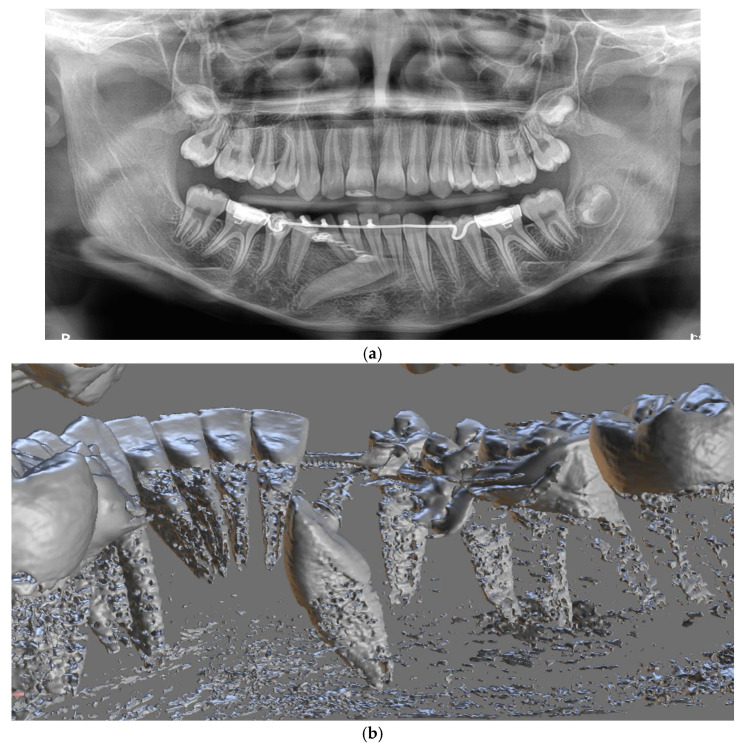

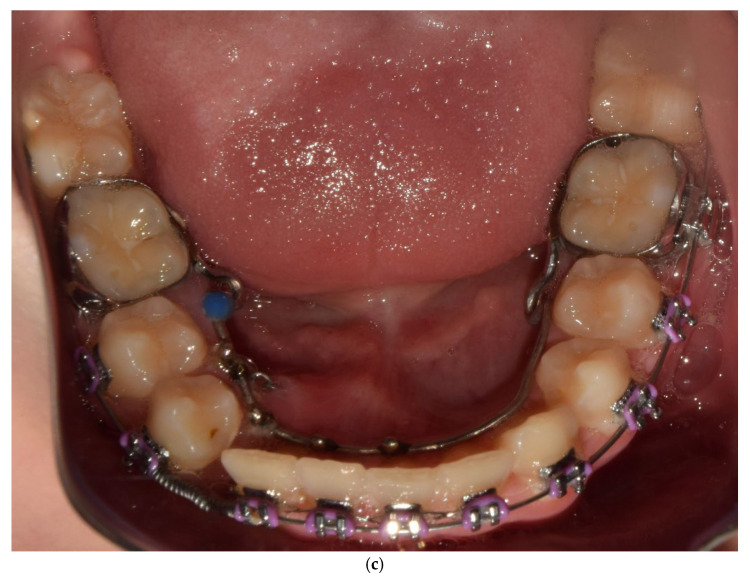
Panoramic radiograph (**a**), CBCT (**b**), and intraoral radiograph (**c**) after insertion of a lingual arch with hooks and surgical exposure of the canine.

The design of the lingual arch with hooks included a semicircular design that indicated the location of the crown of the impacted canine. The lingual arch with hooks included rings that were based on the first lower molars and hooks on which the elastic thread was to be attached to ensure the desired direction of traction of the canine. The entire design was based on the CBCT image and intraoral scans of the patient (Figure 4).

Before cementing the lingual arch with hooks, a surgical exposure of the impacted canine was performed. Under local anaesthesia, the soft tissue and bone immediately above the impacted canine were removed from the lingual side, and after several difficult attempts, an orthodontic attachment with a gold chain was glued to the canine crown. After surgical exposure of the impacted canine, the lingual arch with hooks was cemented, and the gold chain was activated with an elastic thread mounted on the hooks of the lingual arch; next, traction on the canine was initiated, providing gentle forces in the distal-lingual direction.

Following verticalisation and retraction of the tooth beneath the mucosa of the floor of the oral cavity, it was necessary to reposition the tooth into the alveolar process of the mandible. To minimise the risk of root resorption, reduce pain, and accelerate healing during the movement of the tooth into the alveolar process, a local corticotomy with four vertical cuts on the lingual and occlusal side was performed. This procedure was supported by the use of an Er:YAG laser (LightWalker, Fotona, Ljubljana, Slovenia) with settings of 200 mJ, 12 Hz, 2.4 W, a pulse duration of 100 µs, and a conical MSP H14 tip (spot diameter 0.6 mm).

Photobiomodulation (PBM) was applied using a 635 nm diode laser (Lasotronix, Smart ProM, Piaseczno, Poland) with an energy of 10 J per point (20 J/cm^2^) for 100 s per point, delivering a total energy of 20 J per session. Throughout the repositioning process, the direction of movement and the potential risk of resorption were monitored using panoramic radiographs and sectional CBCT scans of the mandible. No complications, including resorption of the canine or the lower incisors, were observed.

**Figure 5:**
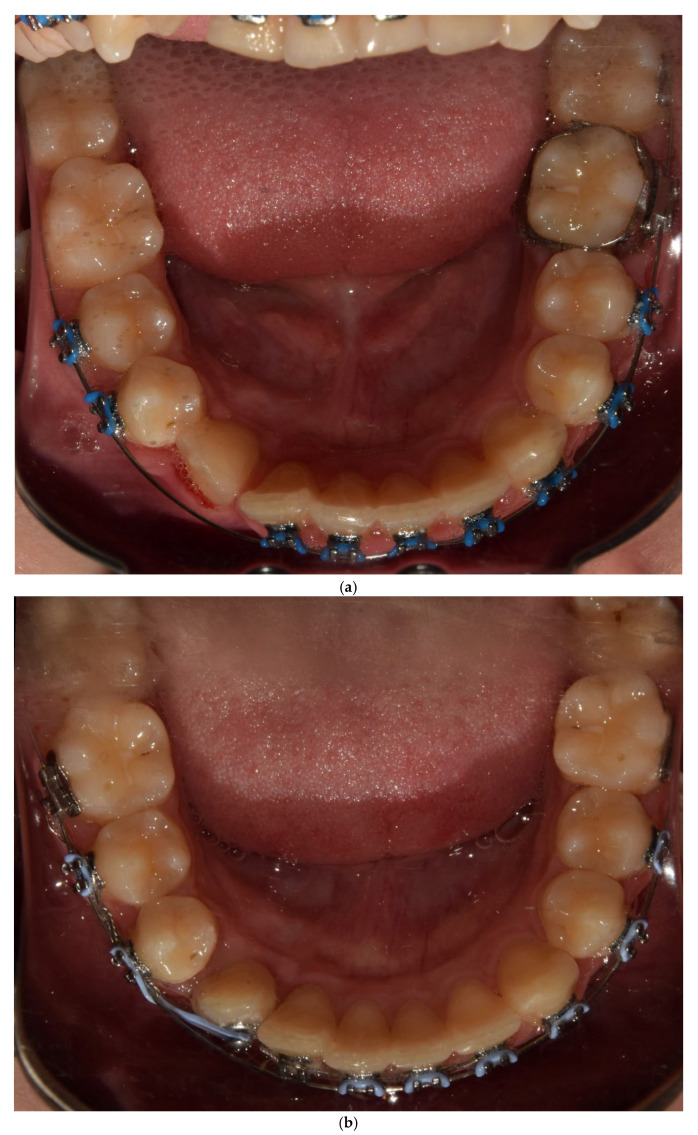
Intraoral photograph of the erupted canine rotated 180 degrees with the lingual arch with hooks removed (**a**). Intraoral photograph during rotation of the canine with an elastic chain (**b**).

**Figure 6:**
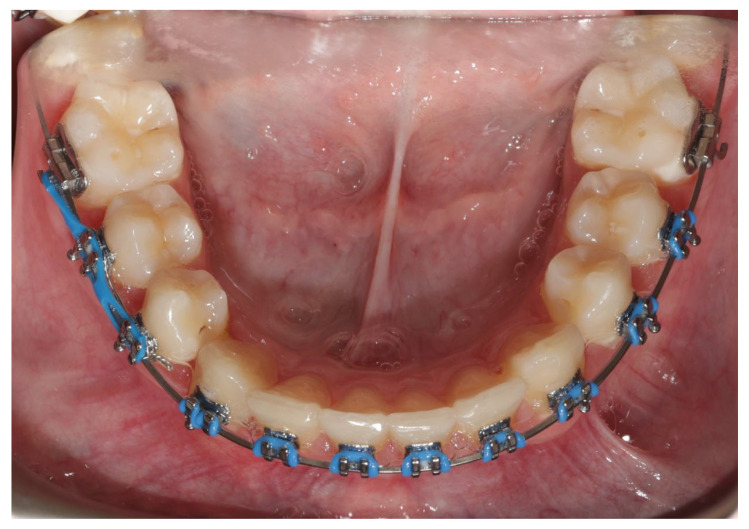
Intraoral photo with the canine inserted into the dental arch.

Following successive activation using gentle orthodontic forces, the canine crown emerged in the anticipated position. However, the crown was rotated by 180°. To correct this, the lingual arch with hooks was removed, and an orthodontic button was attached to the labial surface of the crown (Figure 5). The canine was then rotated to the correct orientation through successive activation using an elastic chain (Figure 6).

**Figure 7:**
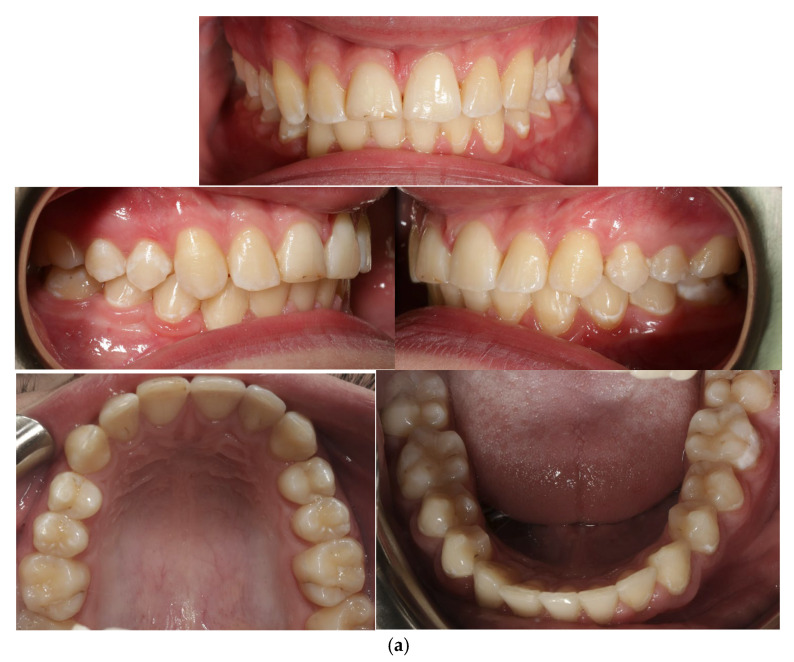

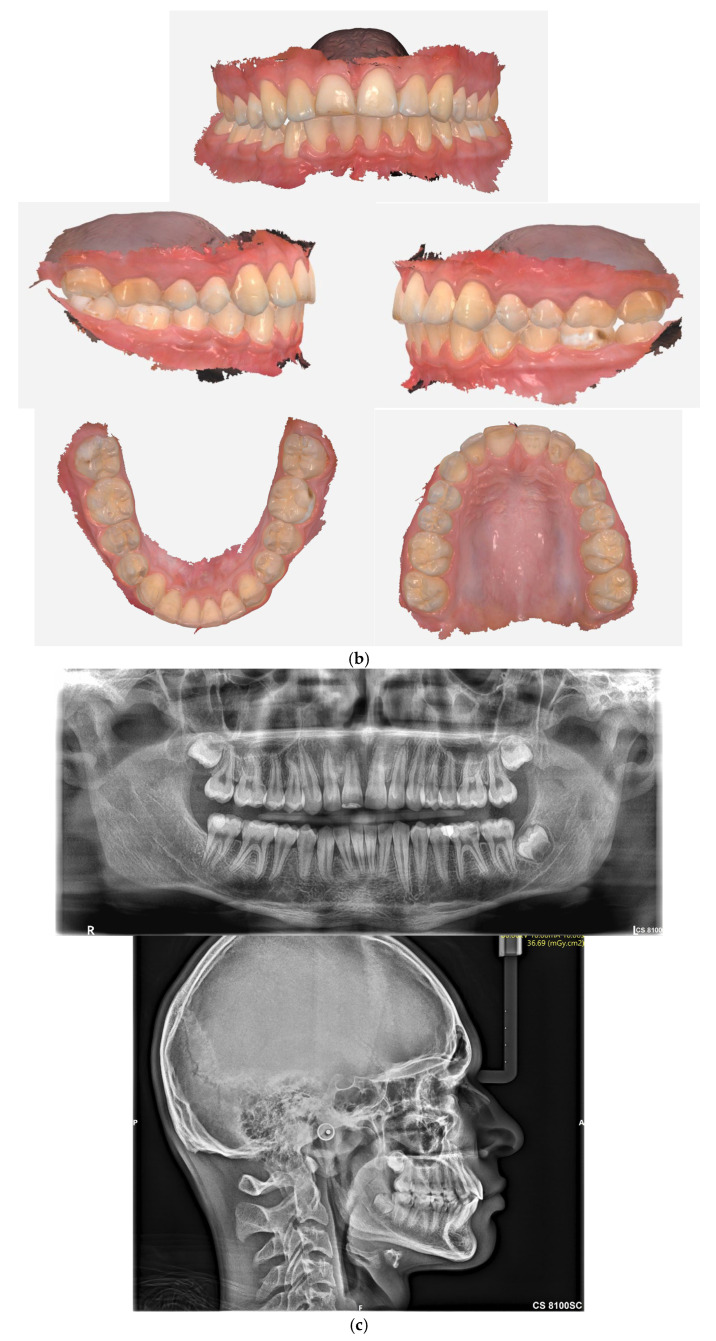
Intraoral photos (**a**), models (**b**), and X-rays (**c**) after treatment.

After all the above-mentioned procedures, the impacted canine was inserted into the dental arch, minimising the risk of canine and incisor resorption, reducing pain, accelerating healing, and fully ensuring the success of the orthodontic treatment. Orthodontic treatment lasted 2 years. The photos show the final situation after inserting the impacted tooth 43 into the arch (Figure 7).

## 3. Discussion

The incidence of impacted mandibular canines is relatively low, but severe impaction is associated with significant clinical dilemmas and challenges [1,18]. Based on the scientific literature, when there is insufficient space for the eruption of the mandibular canine, the most common procedure is surgical extraction or leaving the impacted canine untreated. In the case of leaving the impacted tooth, frequent radiological monitoring is crucial to assess the situation [3]. Management of impacted canines requires a multidisciplinary team consisting of surgeons, orthodontists, and periodontists. It involves a highly complex and close interaction that is essential for optimal treatment. Each case is unique with its own variables and will require proper diagnosis, treatment planning, timing, appropriate surgical exposure techniques, and appropriate orthodontic mechanics to achieve optimal results and reduce the risk of mucogingival problems. Accurate diagnosis using 3D imaging along with technological advances allow a multidisciplinary team of clinicians to carefully plan and execute treatment to ensure the best outcomes for patients [11,19].

Two-dimensional (2D) imaging can provide valuable diagnostic information but presents limitations in accurately determining the position of impacted teeth. Since the advent of cone beam computed tomography (CBCT) in dentistry, it has become a critical diagnostic tool routinely used to evaluate impacted teeth, providing precise information about their position and relationship with adjacent teeth [2,19]. CBCT offers a three-dimensional, high-resolution perspective, surpassing conventional dental radiographs in visualising hard tissues [9,11,13,20]. This technology enhances the clinician’s confidence in determining the position of impacted canines, assessing their contact with adjacent teeth, and identifying any root resorption [21].

In this case, CBCT scans were integrated with STL files obtained via intraoral scanning. Using these digital models, a custom-designed lingual arch with hooks was fabricated to facilitate surgical exposure and orthodontic traction of the impacted canine. The lingual arch incorporated a projection with hooks designed to support elastic thread traction. The device’s shape, size, and thickness were tailored following an analysis of the optimal traction direction, ensuring precise guidance of the canine into the desired position during the initial treatment phase [21].

Surgical exposure of the impacted canine is a prerequisite for orthodontic traction. This procedure involves raising a full-thickness flap, removing the bone covering the crown of the impacted tooth, bonding a gold chain attachment to the crown, and re-approximating the flap to its original position [19]. However, surgical exposure and orthodontic traction often result in prolonged treatment duration, with associated risks such as root resorption and patient discomfort [18]. To address these challenges, local corticotomy may be employed to shorten treatment time and reduce the risk of root resorption while ensuring high-quality orthodontic outcomes.

The goal of corticotomy is to incise the cortical layer of the alveolar bone, inducing localised and temporary osteopenia. Minor stimuli, such as shallow bone incisions, activate the RANK/RANKL system, leading to accelerated bone remodelling—up to 10–50 times faster than normal [22]. Corticotomy-assisted orthodontic treatment (CAOT) is a contemporary approach designed to expedite treatment and mitigate some limitations of traditional methods. Recent studies have demonstrated that selective localised decortication, when combined with orthodontic tooth movement, promotes rapid alveolar bone remodelling, reduces periodontal ligament hyalinisation, eliminates the lag phase in the later stages of tooth movement, and decreases root resorption on the corticotomy side [15,16,23,24].

The use of corticotomy allows for an increase in the volume of the alveolar process bone and periodontium in the case of expansion, while in the case of various types of orthodontic movements, it shortens the treatment time, improves post-treatment stability, contributes to a decrease in the number of relapses, and influences the rapid regeneration of impacted teeth. CAOT is a technique that has many applications in adult orthodontic treatment, as it helps overcome many of the current limitations of conventional treatment, including long duration, potential periodontal complications, and limited tooth movement [15].

To further minimise root resorption, alleviate pain, and expedite healing during tooth movement towards the alveolar process, photobiomodulation (PBM) was applied. A 635 nm diode laser (Lasotronix, Smart ProM, Piaseczno, Poland) was used at an energy of 10 J per point (20 J/cm^2^) for 100 s per point, delivering a total energy of 20 J per session. Low-level laser therapy (LLLT), used as an adjunctive treatment, complements traditional orthodontic methods by potentially reducing treatment time and enhancing overall outcomes. LLLT facilitates alveolar bone remodelling by increasing osteoblast and osteoclast activity. Orthodontic tooth movement is a biological response to external forces. These forces create a tension side and a pressure side. On the pressure side, bone resorption occurs, while on the tension side, bone apposition occurs. LLLT triggers these remodelling processes through its photobiostimulating effects. These effects occur when the tissue absorbs the laser light, which activates an intercellular signalling cascade, increasing cellular metabolism and anti-inflammatory properties [25,26].

It should be noted that the morphology of the mandibular alveolar processes and the higher incidence of severe transmigration of the mandibular canines are factors influencing the treatment prognosis. Late diagnosis of impacted canines may result in root resorption of adjacent teeth and, during traction, also in the resorption of the impacted tooth [7,11].

There are some limitations of such treatment. The first is good patient cooperation in order to precisely expose and stick the abutment and then be able to activate it. The next is the ability to keep the field dry in order to stick the abutment, which is often a big challenge.

Other limitations of the described method are higher costs, technical complexity, and reproducibility in less-equipped clinical settings.

## 4. Conclusions

The digitally designed and 3D-printed lingual arch with hooks enabled precise control over the traction of the impacted and transmigrating tooth. When combined with local corticotomy and laser therapy, this approach effectively minimised root resorption, reduced pain, accelerated healing, and improved treatment outcomes. It can be an alternative treatment instead of extraction. In summary, these procedures successfully restored the occlusal norm by integrating the canine into the arch without complications, offering a potential guide for managing similar cases.

## Data Availability

Data were obtained from Dental Star Specialist Centre of Orthodontics, Krakowska 4/2, 15-875 Bialystok, Poland.
